# Is There Still a Role for Glycoprotein IIb/IIIa Antagonists in Acute Coronary Syndromes?

**DOI:** 10.4021/cr251w

**Published:** 2013-03-08

**Authors:** Loredana Iannetta, Paolo Emilio Puddu, Domenico Cuturello, Angela Saladini, Mariano Pellicano, Michele Schiariti

**Affiliations:** a Department of Cardiovascular, Respiratory, Nephrological, Anesthesiological and Geriatric Sciences, Sapienza, University of Rome, Italy; b Sant’Anna Hospital, Catanzaro, Italy

**Keywords:** GP IIb/IIIs inhibitors, High risk patients, Acute coronary syndromes, Acute ischemia, PCI

## Abstract

The role played by glycoprotein (GP) IIb/IIIa inhibitors has continuously evolved from the initial introduction in mid 90 s until the most recent guidelines for treating acute coronary syndromes, and competed with a wider use of ADP inhibitors and novel anticoagulant drugs, to the extent that they stepped down from class I to class II recommendation in the routine setting of acute coronary syndromes. As a consequence, GP IIb/IIIa use was greatly narrowed. The purpose of this review is to define the roles that GP IIb/IIIa inhibitors may still have in acute ischemic settings by explaining why in high risk patients they might be preferable and/or whether they might be added to ADP inhibitors also emphasizing the underlying mechanistic actions. It is concluded that there might be a more extensive use of GP IIb/IIIa inhibitors in patients presenting with acute coronary syndromes, strictly based on the definition for a high risk procedure: complexity, angiographic characteristics and patient’s risk profile, regardless whether STEMI or NSTEMI. The positive elements one should appreciate in GP IIb/IIIa inhibitors are: efficacy, rapid onset and reversibility of action, absence of pharmacogenomic variability, pharmacoeconomic considerations and the possibility of intracoronary administration.

## Introduction

Platelets are smooth and discoid blood elements lacking several organelles of other cell compartments, but presenting essential structures whereby their role in hemostasis is fully expressed which has the counterpart of being the base for thrombotic events when stimulation is inappropriate [1]. The first step to initiate arterial thrombosis is endothelial injury and exposure of subendothelial matrix glycoprotein (GP) to circulating platelets with ensuing adhesion. Collagen seems to be the most important ligand, even if under specific conditions other molecules, for example von Willebrand Factor (vWF), play a critical role in platelet adhesion [2]. On platelet surface GPs Ia/IIa, Ic/IIa, α_V_β_3_ and Ib/IX mediate platelet adhesion [2]. However, platelets become activated only after they adhere to a site of injury [3]. Biochemical and mechanical mediators cause platelet activation: it seems that in the pathological setting there are upward of 100 biochemical agonists, including ADP, epinephrine, collagen and vWF [3, 4].

Platelet aggregation is mediated by GP IIb/IIIa binding fibrinogen and vWF and other ligands through a transition from a low to a high affinity state for its ligands, bridging platelets together [5]. Although resting platelets have a low affinity for fibrinogen, when they activate can bind more than 40,000 molecules per cell [3]. Antiplatelet therapy has been shown to significantly reduce the risk of serious vascular events in high-risk patients, including those with a prior acute ischemic event and/or ST segment elevation myocardial infarction (STEMI). Long-term antiplatelet agents are key components of secondary prevention after acute coronary syndromes (ACS), including STEMI. However, there might be a critical balance to monitor: any effective antiplatelet regimen may be closely related to increased risk for bleeding, often necessitating discontinuation of treatment and directly impinging on a potentially worse long-term outcome [6, 7].

The role played by GP IIb/IIIa inhibitors has continuously evolved from the initial introduction in mid 90s until the most recent guidelines for treating acute coronary syndromes, and competed with a wider use of ADP inhibitors and novel anticoagulant drugs, to the extent that they stepped down from class I to class II recommendation in the routine setting of acute coronary syndromes [8, 9]. We then review the current role of GP IIb/IIIa inhibitors in acute ischemia and try to explain why in high-risk patients they might be preferable and/or might be added to ADP inhibitors which mostly rely on their underlying mechanism of action.

## Mechanism of Action of GP IIb/IIIa Inhibitors

The wide use of percutaneous coronary interventions (PCI) may induce a thrombotic state by injuring vessels’ walls and by stimulating platelet activation and neo-intimal proliferation. In fact, acute occlusion due to stent thrombosis represented a major event causing acute myocardial infarction, cardiac death and the necessity for a new procedure or coronary by-pass intervention when coronary stents were positioned at the very beginning of their use after failure of balloon angioplasty. Antiplatelet therapy then became standard practice when coronary revascularization procedures were undertaken and aspirin played a pivotal role among these drugs since it inhibited cyclo-oxygenase enzymes, key factors in the platelets’ activation pathways [10-14, 15-18]. Dual antiplatelet therapy ameliorated adverse events related to drugs used during angioplasty [19, 20]. Pre-treatment with aspirin and ticlopidine was found to be very effective, reducing acute intra-stent thrombosis [21]. On the other hand, a two-step strategy, separating diagnostic from interventional times was selected. In fact, in the pre GP inhibitors’ era, dual antiplatelet therapy was done before the patient was admitted to the catheterization laboratory since ticlopidine or clopidogrel required several days or hours before target antiplatelet effects were obtained [15-18].

GP IIb/IIIa inhibitors opened new treatment possibilities because by rapid antiplatelet action they enabled a one-step revascularization strategy, directly downstream in the catheterization laboratory [15-18]. Because of the low affinity for ligands in resting platelets and its increase after platelets are activated, being GP IIb/IIIa the final common pathway of platelet aggregation [4] it soon became the target for specific and rationally elaborated antiplatelet drugs [1].

GP IIb/IIIa are integrins, a large family of adhesion receptors, obligate heterodimers, each one composed of a large extracellular domain, a single pass transmembrane segment and a small cytoplasmic tail [22]. They exist in a low affinity state on cell surface but, upon stimulations mediated by specific intracellular signals, they convert into active state permitting linking to extracellular ligands (inside-out activation) which promotes interaction of intracellular proteins with cytoplasmic tails (outside-in activation) [23]. In the active state, the extracellular domain was shown to switch from a bent to an extended conformation [23]. In presence of calcium, the crystal structure of the extracellular domain is severely bent forming a compact “V” shape and in presence of magnesium integrin assumes an extended conformation: this is the “switchblade hypothesis” [5]. Cytoplasmic proteins that bind to the cytoplasmic tail play a critical role in initiating and propagating the bidirectional signalling events across the integrin [5]. On the other hand, inhibiting GP IIb/IIIa either alone or with α_V_β_3_ receptor attenuates TF-induced prothrombin activation [2]. So GP IIb/IIIa inhibition may offer both antiplatelet and anticoagulant effects [3]. Progression of arterial thrombosis, mediated also by pro-coagulant activity of activated platelets, is thus doubly inhibited by GP IIb/IIIa inhibitors [2].

GP IIb/IIIa is the most abundant integrin on platelet surface and peptides containing the sequence Arginin-Glycin-Aspartate can inhibit interaction between this integrin and fibrinogen [24]. A number of antibodies against platelet GP IIb/IIIa were developed using animal models, particularly dogs. To prevent clearance of platelet with adhered antibodies, the Fc component was cleaved and, to limit immunologic response to the Fab fragments, a mouse/human chimeric antibody was developed called abciximab [24]. Free plasma abciximab is cleared from circulation in minutes while drug-platelet complexes persisted up to one week depending on platelet turnover [4]. Eptifibatide and tirofiban, two small-molecules belonging to GP inhibitor class, are respectively a peptide-mimetic linking Arginin-Glycin-Aspartate sequence with a plasma half-life of 2.5 hours and a non-peptide tyrosine derivative blocking the same site with plasma half-life of 2 hours [2].

GPI must maintain more than 80% of receptor occupancy to achieve sufficient therapeutic efficacy [4]. These drugs can be administered only intravenously because, if given orally, there is a paradoxical fibrinogen binding effect related to plasmatic levels. In fact, during high plasma concentration, sufficient quantities can successfully inhibit platelets competing with physiological ligands, while during “troughs” GP IIb/IIIa would remain in extended activated state, exposing binding site to physiological agonists [25].

Thrombocytopenia and major bleeding are the more frequent complications associated with this class of drugs [26, 27]. Security and efficacy of these agents have been widely demonstrated and it should be considered that GP inhibitor induced thrombocytopenia is less related to increased risk of clinical complications than thrombocytopenia secondary to other causes (for example hematological, drug induced, septical or in relation to low output states) [26, 27].

## Glycoprotein Inhibitors: A Bit of History

Safety and efficacy of bolus (0.25 mg/kg) followed by infusion (10 µg/min) of abciximab were evaluated in EPIC trial as early as 1994, nevertheless it was associated to high bleeding risk and to thrombocytopenia and immune-mediated hypersensitivity [28]. The ISAR-REACT 2 trial evaluated GP inhibition in adjunct to thyenopiridine in high risk patients: abciximab administration was associated to better outcomes relying to troponin levels in patients with acute coronary syndromes without ST-segment elevation [29]. The use of tirofiban (10 µg/kg bolus followed by 0.15 µg/kg/min infusion) during PCI was evaluated with controversial results in TARGET (compared to abciximab) and RESTORE (compared to heparin alone) trials in 1997 - 2001 [18, 30].

Firstly Schneider proposed a high dose bolus tirofiban (25 µg/kg bolus) to improve efficacy of tirofiban during PCI, obtaining antiplatelet effects similar to abciximab with lower costs [31-33]. Similar to tirofiban, also eptifibatide dosing was adjusted since its initial employment, observing an optimal antiplatelet effect of the double bolus (two 180 µg/kg boluses 10 min apart and a continuous infusion at 2.0 µg/kg/min for 18 - 24 hours), as assessed in ESPRIT trial [34]. The safety and efficacy of high-dose bolus tirofiban were reported in comparison with abciximab [35-41]. Several studies compared eptifibatide to abciximab [42-44], whereas only SANTISS directly compared high-dose bolus tirofiban with double bolus eptifibatide and the superiority of tirofiban was shown [33].

## When and why Administer GP Inhibitors (Based on European Guidelines)?

Current European Society of Cardiology guidelines about patients without ST segment elevation myocardial infarction (NSTEMI) consider upstream use of GPIIb/IIIa inhibitors in active ongoing ischemia among high risk patients or when double antiplatelet therapy is unfeasible. It is reasonable to use GP inhibitors in patients undergoing PCI based on angiographic results such as presence of a thrombus or troponin elevation, previous treatment with P2Y12 inhibitors, patient age and bleeding risk. Nevertheless, it is reasonable to combine GP inhibitors with dual antiplatelet therapy in patients undergoing high risk PCI and without high bleeding risk. In association with novel anticoagulant drugs such as bivalirudin GP IIb/IIIa inhibitors are not recommended because of worse outcome [9].

In STEMI, the abovementioned guidelines, declare that the role of GP inhibitors during primary PCI and the era of novel antiplatelet drugs, particularly prasugrel and ticagrelor, is not well defined. It is reasonable to use them in STEMI, similar to NSTEMI, as bailout therapy when there is angiographic evidence of a large thrombus, slow-flow or no-reflow or other particular cases. However, they are not recommended when bivalirudin is used [8]. As a consequence, in STEMI patients, periprocedural antithrombotic medication in primary PCI stepped down GP inhibitors from I to II class of recommendation, whose level of evidence was judged A for abciximab and B for high double bolus dose eptifibatide or high bolus dose tirofiban [8].

Intra-venous route should remain the standard strategy, although intra-coronary administration may be considered [8].

## GP Versus ADP Inhibitors

ADP-induced platelet activation involves two receptors: P2Y1 and P2Y12. Separate inhibition of one of the formers may result in a significant inhibition of platelet aggregation, although P2Y12 plays the major role. P2Y1 is coupled to a G_αq_ protein, triggering the release of calcium from internal stores, inducing platelet shape change and weak, transient ADP induced aggregation, but on the other hand it is a crucial factor for ADP or collagen induced platelet activation [45]. P2Y12 is coupled to G_αi2_ protein, a critical component of the activation pathway of GP IIb/IIIa [45]. Thienopyridine drugs, such as ticlopidine, clopidogrel and prasugrel and non-thienopyridine ticagrelor bind and inhibit P2Y12 receptor and therefore platelet aggregation [46]. Periprocedural antiplatelet therapy including these drugs has been wholly questioned, particularly considering wider use of bivalirudin as anticoagulant support for PCI [47].

Wider use of higher loading dose of clopidogrel (600 mg) and the birth of new antiplatelet drugs inhibiting ADP receptor such as prasugrel and ticaglelor, definitely narrowed GP IIb/IIIa inhibitors use [8, 9, 48-51]. In STEMI patients undergoing primary PCI bivalirudin or unfractioned heparin plus GP inhibitors (600 mg loading dose of clopidogrel) was associated with a lower rate of 30-days major adverse outcomes [52].

It is of paramount importance to balance bleeding risks with risk of recurrent ischemic events. Even if some authors reported a not increased major bleeding rate in STEMI patients undergone to rescue angioplasty and GP IIb/IIIa inhibitor administration, there are discordant results [53]. Some genetic polymorphism of CYP2C19 loss of function, an hepatic enzyme contributing to the metabolism of many clinically relevant drugs, inclusive of clopidogrel, are related to drug resistance [54-57]. Recent studies showed a suboptimal platelet inhibition lasting the first 2 hours after prasugrel administration which might be obviated co-administering a bolus of GP inhibitors intravenously [58]. Therefore in the setting of STEMI patients the role of GP IIb/IIIa inhibitors is yet important, considering that intracoronary administration showed higher local receptor occupancy and improved microvascular perfusion [59-61].

Pre-hospital administration of GP IIb/IIIa inhibitors, particularly tirofiban, given very early after symptoms onset, seems associated to better revascularization outcomes [62]. Even in NSTEMI patients early administration of GP IIb/IIIa inhibitors was associated with beneficial effects and it was continued until after procedure in patients undergoing PCI [63]. On the other hand, when novel anticoagulant drugs such as bivalirudin are administered, it was shown that use of GP IIb/IIIa inhibitors is not recommended, particularly when renal function is damaged [64].

A very recent study proposed GP IIb/IIIa inhibitors as bridging therapy for patients with drug eluting stents undergoing surgical procedures [65].

## Conclusions

GP IIb/IIIa inhibitors may still be considered effective drugs in STEMI patients and in high risk NSTEMI patients undergoing PCI. They inhibit the final common pathway of platelet aggregation, downstream to the ADP pathway regulated by P2Y12 inhibitors. They have a prompt and effective antiplatelet effect compared to ADP inhibitors and they are not influenced by patient genotypes.

Compared to thienopyridines, that have a non-reversible antiplatelet effect and a less rapid onset action, novel non-thienopyridines, cangleor and ticagrelor seem to have a promising wider use [27]. However, no head to head comparisons between these non-thienopyridines and GP IIb/IIIa inhibitors were performed and it is not probable any will be performed in the near future. Thus, a non inferiority of non-thienopyridines may not be concluded. On the other hand no ADP inhibitor showed a prompt platelet inhibition compared to GP IIb/IIIa inhibitors.

Guidelines try in general to address routes on the basis of “one concept fits all” which is definitely quite impossible in clinical conditions such as acute myocardial infarction, whether STEMI or NSTEMI, rather presenting a spectrum than a clear-cut pathology. Accordingly, not all patients’ subsets may fit the currently available guidelines [8, 9]. It is our opinion that actual data, as reviewed here, may sustain a more extensive use of GP IIb/IIIa inhibitors in patients presenting with acute coronary syndromes, strictly based on the definition for a high risk procedure: complexity, angiographic characteristics and patient’s risk profile, regardless whether STEMI or NSTEMI. The positive elements one should appreciate in GP IIb/IIIa inhibitors are: efficacy, rapid onset and reversibility of action, absence of pharmacogenomic variability, pharmacoeconomic considerations and the possibility of intracoronary administration.
